# Traveling Memoranda from the Editor

**Published:** 1836

**Authors:** 


### II.—ORIGINAL.

---

### THE WESTERN JOURNAL.

---

*E Sylvis Nuncius.*

---

CINCINNATI, JANUARY 1st, 1837.

---

### I.—Traveling Memoranda.—From the *Editor.*

*Nashville Tennessee, July 21st,* 1836.—From Huntsville to this city, by way of Pulaski and Columbia—a distance of 114 miles—the road traverses the upper trunks and branches of Elk, Duck and Harpeth *rivers,* neither of which is of great volume. Throughout the whole distance it passes over a lime-stone formation. For the first 25 miles, this stone is the same with that about Huntsville, and constituting the basis of *Monte Sano* in its neighborhood. It is in thick massive strata, of an ash color, and containing but few organic remains. Abounding in rents and caverns, it collects the rains into copious, perennial springs. The traveler observes this stone to graduate into a bluish lime-stone, more opulent in petrifactions, and in thinner laminæ—almost exactly resembling that found along the Ohio, from Maysville to Louisville. The moment he has fairly gotten upon this variety of the great western calcarious formation, he observes a change in the kind of vegetation, and finds himself in the midst of the trees and herbaceous plants of Kentucky, Ohio, and Indiana. Such is the invariable relation between soils and their vegetable productions. He remarks still farther, that the number of copious perennial springs has diminished; and, I should have added above, that the red soil with its framgents of chert or petrosilex, is replaced by a yellowish loam, much less abundant in those silicious reliquiæ of the rocks which have been decomposed, because the blue lime-stone, contains fewer embedded flints than the ash colored. Elk, Duck and Harpeth rivers, seem like those of Kentucky, to become nearly exhausted of water in the summer, to have a sluggish current, and narrow alluvial bottoms. The whole surface of the country through to this place, is indeed, rolling, dry and comparatively free from the ordinary sources of malaria. Still, in the immediate neighborhood, of the small and lagging rivers, exhalations of this kind arise. In passing over this region, the traveler who belongs to the banks of the Ohio, is struck with the fact, that the amount of loam and soil on the surface of the rocky strata, is much less than in Kentucky, and far less than in Ohio, Indiana and Illinois. What causes may have determined a more rapid disintegration of the rocks further to north, and a consequent deeper bed of earthy matter, is not perhaps easily known, when they so nearly resemble each other in constitution and constituents.—Throughout this part of Tennessee, the rocks are very often seen projecting their weather beaten edges through the soil. Their constant decay, of course, keeps up its fertility, but still, on the whole, they lie too near the surface.

The spot on which this city is built, presents the *maximum* of this *deficiency*. Let the reader imagine, on the left or southern bank of the Cumberland river, an undulating plain, terminating abruptly at the water's edge, and rising from 100 to 150 above the surface of the stream, with so much of the soil removed, that even shade trees cannot, without blasting the rocks, be cultivated in the streets, and he will have a *prima facie* idea of the site of Nashville. Let him, then, fancy the exposed strata to abound in organic remains, and to be covered, wherever the trees have not been cut down, with red cedar, (*Juniperus Virginiana*) and his conception of this picturesque spot, as far as its natural features are concerned, will be nearly complete. To perfect it, however, he must ascend the beautiful, regular, oval eminence, denominated from the standing grove, Cedar Hill, and take the panoramic view, which can there be had, when he will at once come to the conclusion, that Nashville is not only picturesque and beautiful in its natural scenery, but, notwithstanding the presence of a winding river, enjoys all the geological elements of a healthy situation.

Just below the city there is, it is a true, ravine opening into the bed of the river, which in high floods is filled with water to a great depth. But its shortness and narrowness, render it comparatively innocent as to the production of malaria. In the bottom of this ravine, quite adjoining the city, is a

SULPHUR WELL, slightly impregnated with salt. It is an excellent water for invalids, and, what I regard as remarkable, is much frequented by the *people of the city*. High, however, as they esteem its waters, the hand of taste has thrown about it, scarcely any of those decorations of which it is susceptible, and which would so greatly contribute to the attraction of strangers. The reason assigned for this neglect, is the repeated inundations to which the spot is liable; but these do not occur during the watering season, and, it would not be difficult, by a moderate expenditure, in fences that could not float away, and in the cultivation of trees and shrubs and grass, that would bear submersion, to increase the natural beauties of the spot, till, with due arrangements in the city for the accommodation of those who are wont to visit watering places, great numbers could be annually drawn hither.

LUNATIC ASYLUM.

The state of Tennessee has erected a hospital for insane persons, in this city. Patients have not been introduced into it yet. Its situation is retired, and at the same time sufficiently conspicuous. The benevolent mind cannot but long for the time, when every state in the West and South, will follow the noble example of Kentucky, and construct at least one capacious and commodious edifice for the insane. On this subject, physicians might exert a great influence, and they owe it to themselves and the dignity of the profession, not less than to the interests of humanity, to put forth the power, which now lies dormant in them.

### Physicians.

The profession in Nashville, notwithstanding the recent death of the estimable Dr. Roane, is respectable. Drs. Robertson, McNairy and Overton are among the old and eminent physicians of Tennessee; but the majority of the physicians of the city are young men. They are not organized into a society for mutual improvement; but they constitute the nucleus, (and this is almost the entire crystal) of the State Medical Socity, which holds, or ought to hold, its annual meeting in this city. The society makes a yearly publication of its proceedings, in connexion with a professional address from one of its members; but notwithstanding a considerable array of names, as officers and committee men, its meetings, on the whole, like those of other western state societies, are but thinly attended. The annual discourse of last spring, was by Dr. Buchanan of Columbia, on the Medical Topography and Diseases of Middle Tennessee, and appears on a slight inspection to abound in new observations. Much credit ought to be awarded, to the few physicians who labor to maintain the organization of this state society, and render it useful in the elevation of the profession.

### University of Nashville.

Here we find Dr. Troost, an ardent, affable, unassuming old gentlemen—old in years and science, but young and simple in his feelings and manners. I have just returned from one of his geological lectures—its topic the lime-stones of Tennessee. His acquaintance with the subject was manifestly most intimate and profound; but the taste of his class appeared to be different from his own; and I should not judge that it will ever turn out a successor to supply his place. The collections of Dr. Troost, especially in organic remains, are very great; but the arrangement of them is far better in his own philosophical mind, than in the various apartments into which they have been accumulated. Like most other enthusiasts in science, Dr. Troost is less intent on the pursuit of gain than glory. At his own expense, he makes many costly publications, illustrative of the fossil zoology of the state which has adopted him; and thus, by an expenditure from his very moderate salary, contrives to build up a scientific reputation in his native Europe. Happy, indeed, would it be for the country, if his example could awaken a general emulation, among its physicians, professors, and men of fortune.

The Trustees, by the advice of the learned and very talented President Lindsley, have taken a sort of lead in the West, in establishing a professorship of *Anatomy* and *Physiology*, as branches of study for under graduates. Dr. Jennings, one of the most able and enterprising physicians of the city, has been called to this chair; and seems bent on throwing around it the interest, with which it is certainly susceptible of being clothed. It is easy to perceive, that in all this, there is an ulterior object. Other professorships of medicine will in due time be added, and the University of Nashville, at no distan day, present to the profession a MEDICAL DEPARMENT. And why should it not? In Italy, almost every little principality has its medical school; and, along our own seaboard, from Long Island Sound to the Chesapeake Bay, a distance less by one-third, than the length of the state of Tennesse, there are six schools, and another seriously projected in Philadelphia. This state requires as many physicians as could be *well* educated in one institution; and Nashville is favorably situated for such an establishment. Moreover, the adjoining parts of Virginia, the Carolinas, Georgia and Alabama would contribute to the supply of pupils. I say let he project *go ahead.*

*Graham's Springs, Harrodsburgh, Kentucky, July*, 27th. From Nashville to this place the road lies mainly over a hilly country. In crossing Barren, Green and the Rolling Fork of Salt River, a decided elevation is attained, and the Cumberland mountains, or rather spurs of them, are often seen to the right. The limestone disappears, or is alternated with shale and sandstone, which often exhibit appearances of coal and iron, with numerous fragments of buhr millstone. As we approach this place nothing but lime-stone appears at the surface. On this road, as from Huntsville to Nashville, a change in the geology is attended with a change in the botany. The shale and free-stone formation, bears several kinds of oak, not found on the calcarious soils, also chesnut, persimon and sassafras trees in abundance. On reaching the limestone lands, of the basin of Salt river and the Kentucky, the sugar tree, buckeye, blackwalnut, hackberry, blueash and pawpaw, with an undergrowth of eupatorium (not the *perfoliatum*) such as cover the calcarious ridges of middle Tennessee, re-appear, and remind the experienced eye, of the fertility of which they are the unerring *indiciæ*.

THE SPRINGS.

The stage brought us to this place after dark. Its hundred lights, distributed throughout the immense group of cottages, and illuminating the surrounding shrubbery, gave it the appearance of fairy land. I have since found on a general and particular examination by day light, that Dr. Graham, has in fact made a very heavy expenditure on this spot, and, on the main, with excellent judgment, and very little bad taste. His attempt to form a small artificial lake, has not been successful for the want of the essential ingredient, water; which on this summit level between the branches of Salt river and the Kentucky, is a scarce article. The readers of the Western Journal, at least of its 2d volume, need not be told, that the predominent ingredient in the water of these springs, is epsom salt,—sulphate of magnesia,—or, that the lime-stone of this region abounds in that substance. Extensive as are the accommodations, new comers are daily turned off in disappointment. Of the visitors, the chief I find, are Kentuckians and Mississippians. Such popularity speaks well both for the springs and their obliging, intelligent, and enterprising proprietor.

*Lexington, August 8th.*—Here we are, in the early metropolis of the West. The spot where the first school for medical instruction in the interior of the continent, was established; where the first medical degree was conferred west of the mountains; and to which a very large number of the most intelligent and active physicians of the Valley of the Mississippi, turn their thoughts and affections, as the seat of their *alma mater*.

### Medical Societies.

The *Lexington Medical Society* was incorporated many years ago, and has been the seat of numerous animated debates; but we Americans, get tired of our play things, and seek for new ones. Use and age, generate no endearments; and hence the physicians of Lexington, embracing several or all the medical professors of the University, have lately had themselves incorporated, under a new charter, which styles them the *College of Physicians and Surgeons*. I had the opportunity of attending one of the meetings of this society, (of which the learned professor Caldwell is the president) and found that a part of their exerecises, consists in monthly reports on the prevalent diseases of the city, and another part, in the report of individual cases, with speculations on their pathology and treatment. Both practices are worthy of praise; and should be imitated by the physicians of all our principal towns.

### Lunatic Asylum.

The noblest institution in this city, is the hospital for insane persons, established by the state of Kentucky; and admirably managed, by a number of intelligent and respectable citizens of Lexington. On a visit to it, I have been gratified to meet among its managers, not a few of the same gentlemen, whom I used to meet ten years before, when acting as one of its medical attendants. This continuance in office, is as it should be. No charitable institution can flourish, under a perpetual change of managers. Even the same kind-hearted steward and matron, were there still; and the attending physician, Dr. Theobald, whose urbanity and experience are what the situation requires, has been for several years devoted to the duties of his office. The accommodations, afforded in this establishment, are extensive and well arranged; the warming and ventilation are effected by proper means, cleanliness, quiet and order, are obvious in every part; and I feel it quite a duty to recommend the establishment, to the friends and physicians of the insane, in every western state, which has not yet erected a similar edifice, and brought its internal administration to an adequate degree of perfection.

